# Azole Resistance and *cyp51A* Mutation of *Aspergillus fumigatus* in a Tertiary Referral Hospital in Taiwan

**DOI:** 10.3390/jof8090908

**Published:** 2022-08-26

**Authors:** Tsun-Hao Hsu, Po-Yen Huang, Yun-Chen Fan, Pei-Lun Sun

**Affiliations:** 1 Department of Dermatology, Chang Gung Memorial Hospital, Linkou Branch, Taoyuan 333423, Taiwan; 2 Division of Infectious Diseases, Department of Internal Medicine, Chang Gung Memorial Hospital, Linkou Branch, Taoyuan 333423, Taiwan; 3 College of Medicine, Chang Gung University, Taoyuan 333323, Taiwan; 4 Research Laboratory of Medical Mycology, Chang Gung Memorial Hospital, Linkou Branch, Taoyuan 333423, Taiwan

**Keywords:** *Aspergillus fumigatus*, azole resistance, susceptibility, *cyp51A*, TR_34_/L98H, mutation, resistance, Taiwan

## Abstract

Azole resistance in *Aspergillus fumigatus* has increasingly been reported worldwide. Its major mechanism of resistance is mediated by mutations in *cyp51A*. The objective of this study was to test the antifungal susceptibilities of *A. fumigatus* isolates from Chang Gung Memorial Hospital (CGMH), the largest tertiary referral hospital in Taiwan, and to investigate *cyp51A* mutations in azole-resistant strains. *A. fumigatus* isolates preserved in the Research Laboratory of Medical Mycology of CGMH from 2015 to 2021 were used. Antifungal susceptibility testing was performed using the YeastOne^TM^ method. Isolates with high minimal inhibitory concentrations (MICs) against antifungals were further tested using the Clinical and Laboratory Standards Institute (CLSI) broth microdilution method. Mutations in the *cyp51A* in azole-resistant strains were detected by Sanger sequencing. The overall prevalence of azole-resistant isolates was 1.77% (two out of 113 isolates). The two azole-resistant strains had tandem repeats (TR) in the promoter region and mutations in the *cyp51A* gene (TR_34_/L98H and TR_34_/L98H/S297T/F495I). One strain showed intermediate susceptibility to voriconazole, and its Cyp51A protein had five amino acid substitutions (F46Y/M172V/N248T/D255E/E427K). TR_34_/L98H and TR_34_/L98H/S297T/F495I are the most prevalent *cyp51A* mutations in Taiwan, mediating azole resistance based on current publications and our results. YeastOne^TM^ was validated as a rapid tool for the antifungal susceptibility test; however, further confirmation by CLSI should be considered when MIC values of voriconazole, posaconazole, and amphotericin B are close to the clinical breakpoints or ecological cutoff values.

## 1. Introduction

*Aspergillus* is a genus of ubiquitous saprophytic fungi and is an important opportunistic pathogen in humans. The main route of infection is the respiratory tract through inhalation of fungal conidia [1]. The disease spectrum varies depending on the underlying immune status of the host, and the coincidence of acute bronchopulmonary allergy, severe asthma with fungal sensitization, allergic fungal rhinosinusitis, *Aspergillus* bronchitis, chronic pulmonary aspergillosis, and invasive aspergillosis [1,2].

*Aspergillus fumigatus*, *A. flavus*, *A. terreus*, *A. niger*, *A. nidulans*, and *A. versicolor* are important pathogens of the genus *Aspergillu*s [3]. *A. fumigatus* is the predominant species causing disease in humans [4,5]. Azole resistance in *A. fumigatus* isolates is increasingly reported worldwide [6]. Extensive use of azole fungicidal agents in agriculture has been proven to be associated with azole-resistant isolates of *A. fumigatus* from an environmental source [7,8]. The molecular mechanisms of azole resistance have been extensively investigated in the past decades. One of the major mechanisms of resistance is mediated by mutations in the *cyp51A* gene, which encodes a key enzyme in the biosynthesis of fungal ergosterol. Mutation in the coding region results in the production of Cyp51A with low affinity to azole antifungals [9]. This resistance is exaggerated when there are tandem repeats (TR) in the promoter region of the *cyp51A* gene, which enhances the expression of Cyp51A and the translation of the mutated protein [10]. The 34-bp TR and 46-bp TR within the promoter region with add-on amino acid substitutions were recognized as the predominant resistance mechanisms in environmental *A. fumigatus* strains [11,12]. The prevalent mechanisms were TR_34_/L98H and TR_46_/Y121F/T289A, which conferred resistance or decreased susceptibility to multiple-azoles [13,14,15].

The first azole-resistant *A. fumigatus* in Taiwan was reported by Wu et al. in 2015. In their report, three azole-resistant *A. fumigatus* were identified in 38 isolates from two university hospitals, all of which carried TR_34_/L98H mutations in c*yp51A* [16]. Later, the same group launched a multicenter study that tested 375 *A. fumigatus* isolates from 11 hospitals collected from 2011 to 2018. Azole resistance was detected in 19 isolates (5.1%), and *cyp51A* mutations included TR_34_/L98H, TR_34_/L98H/S297T/F495I, and TR_46_/Y121F/T289A [17]. Chang Gung Memorial Hospital (CGMH) is the largest tertiary referral hospital in Taiwan, with about 3700 beds and 300 ICU beds. The isolates in this study are representative of Northwestern Taiwan.

In this study, we assessed clinical *A. fumigatus* isolates from the CGMH Linkou Main Branch from 2015 to 2021 and aimed to evaluate their susceptibility to different antifungals and examine the *cyp51A* mutations in azole-resistant isolates.

## 2. Materials and Methods

### 2.1. Preparation and Molecular Identification of Fungal Isolates

Fungal isolates preserved and identified as *A. fumigatus* in the Research Laboratory of Medical Mycology of CGMH from 2015 to 2021 were used in this study. The fungal isolates were subcultured on potato dextrose agar at 25 °C for 7 days. Morphological identification was performed by examining the macroscopic and microscopic features, followed by molecular identification for the isolates that could not be identified solely by morphological characteristics. Fungal genomic DNA was extracted using a Smart LabAssist (TANBead^TM^, Taoyuan City, Taiwan) automatic DNA extraction system. Internal transcribed spacers of ribosomal DNA (*ITS*) and the β-tubulin gene (*benA*) were amplified and sequenced. The calmodulin gene (*CAL*) was also used for identification. The primer pairs used were ITS1 (5′-TCCGTAGGTGAACCTGCGG-3′) and ITS4 (5′-TCCTCCGCTTATTGATATGC-3′) for *ITS*; Bt2a (5′-GGTAACCAAATCGGTGCTGCTTTC-3′) and Bt2b (5′-ACCCTCAGTGTAGTGACCCTTGGC-3′) for *benA*; and CMD5 (5′-CCGAGTACAAGGAGGCCTTC-3′) and CMD6 (5′-CCGATAGAGGTCATAACGTGG-3′) for *CAL*. The PCR conditions for each DNA segment were as previously described [18,19]. The sequences of the isolates were used as queries in the BLAST search against the NCBI GenBank database (https://blast.ncbi.nlm.nih.gov/Blast.cgi) (accessed on 1 March 2022).

### 2.2. Antifungal Susceptibility Testing

The Sensititre YeastOne broth microdilution system, YO10 panel (YeastOne^TM^), was used for antifungal susceptibility testing and azole resistance screening. The minimum inhibitory concentrations (MICs) of 5-flucytosine (5FC), posaconazole (POS), voriconazole (VRC), itraconazole (ITC), fluconazole (FLC), amphotericin B (AMB), and the minimum effective concentrations (MECs) of anidulafungin (AFG), micafungin (MFG), and caspofungin (CAS) were determined according to the manufacturer’s instructions. The MIC ranges, MIC_50_, and MIC_90_ values were determined. If the isolates had MIC value(s) of POS, VRC, or ITC ≥1 μg/mL, or AMB ≥4 μg/mL according to the YeastOne^TM^ method, their MICs values were further determined by the broth microdilution method M38 3rd Ed. published by the Clinical and Laboratory Standards Institute (CLSI) [20]. The MICs values for isavuconazole (ISA), POS, VRC, ITC, FLC, and AMB were determined according to the CLSI protocol. All antifungal drugs were purchased from Sigma-Aldrich^®^, and the concentration ranges were as follows: ISA (0.008–4 μg/mL), POS (0.031–16 μg/mL), VRC (0.031–16 μg/mL), ITC (0.031–16 μg/mL), FLC (0.125–64 μg/mL), AMB (0.031–16 μg/mL). *Candida parapsilosis* ATCC 22019, and *Candida krusei* ATCC 6258 were used as the quality controls. Conidia were counted using a hemocytometer and adjusted to a final inoculation size of 0.4–5 × 10^4^ CFU/mL. MIC endpoints were determined using a reading mirror after 48 h of incubation at 35 °C and indicated by a 100% inhibition of growth compared with the drug-free growth control wells for all azoles and AMB. Each isolate was tested twice to verify the consistency.

### 2.3. Detection of cyp51A Mutations in Azole-Resistant Isolates

The analysis of *cyp51A* mutations was performed for isolates with high MIC values for the azole antifungals, as confirmed by the aforementioned methods. The entire *cyp51A* coding region and its promoter region were amplified by PCR. Primers used for the promoter region were TR34-F [5′-TAATCGCAGCACCACTTCAG-3′] and TR34-R [5′-GCCTAGGACAAGGACGAATG-3′]. The primers used for *cyp51A* were CYP1-L [5′-CACCCTCCCTGTGTCTCCT-3′], CYP1-R [5′-AGCCTTGAAAGTTCGGTGAA-3′], CYP2-L [5′-CATGTGCCACTTATTGAGAAGG-3′], CYP2-R [5′-CTTGCGCATGATAGAGTGA-3′], CYP3-L [5′-TTCCTCCGCTCCAGTACAAG-3′], and CYP3-R [5′-CCTTTGAAGTCCTCGATGGT-3′] [21,22]. The sequences of these isolates were queried against the Fungal Resistance Database (FunResDB https://sbi.hki-jena.de/FunResDb) (accessed on 1 March 2022). to detect mutations and tandem repeats in the promoter region of *cyp51A*.

### 2.4. Ethics Statement

This study was approved by the IRB of Chang Gung Medical Foundation (approval number 202200679B0 obtained on 21 February 2022) Patient consent was waived by the IRB.

## 3. Results

### 3.1. Fungal Isolates

A total of 114 isolates identified as *A. fumigatus* were used in this study. One isolate was later identified as *A. flavus* based on its ITS and *benA* sequences and excluded. Other isolates showing slow growth rates, atypical colony colors, or slow sporulation were all confirmed to be *A. fumigatus* based on their sequence characteristics. The final number of isolates enrolled in this study was 113. All 113 isolates were isolated from clinical specimens including sputum (*n* = 58), bronchial lavage (*n* = 39), wound (*n* = 10), deep tissue (*n* = 4), and cornea (*n* = 2).

### 3.2. Antifungal Susceptibility Testing

**Figure 1:**
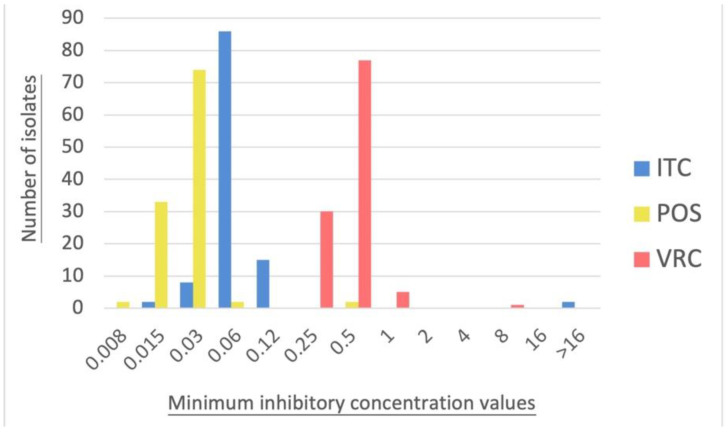
The distribution of the minimum inhibitory concentration values of itraconazole (ITC), posaconazole (POS), and voriconazole (VRC), as determined using the YeastOne^TM^ method. The *x*-axis represents the minimum inhibitory concentration values, and the *y*-axis represents the number of isolates.

The MIC values of the antifungals determined using YeastOne^TM^ are shown in Table 1. All echinocandins showed very low MEC values for all *A. fumigatus* isolates (AFG ≤ 0.015 μg/mL, MFG ≤ 0.008 μg/mL, CAS ≤ 0.008–0.06 μg/mL). The MIC values for 5FC were very high (MIC_50_ and MIC_90_ ≥ 64 μg/mL). The MIC values of AMB ranged from 1 to 4 μg/mL, with both MIC_50_ and MIC_90_ = 2 μg/mL. Three isolates (CGMHD 1497, CGMHD 1524, CGMHD 2417) had higher MIC values for AMB at 4 μg/mL (2.7%). For azoles, the MIC values of FLC were extremely high (up to ≥256 μg/mL), indicating the intrinsic resistance of *A. fumigatus* to this drug. The MIC values of ITC, POS, and VRC showed a normal distribution (Figure 1). Three isolates had MIC values of 1 μg/mL for VRC (CGMHD 0641, CGMHD 0744, CGMHD 2261) and one with 8 μg/mL (CGMHD 1652). Two isolates showed high MIC values of >16 μg/mL for ITC (CGMHD 1652 and 2261). For these seven isolates, which showed high MICs for AMB, VRC, or ITC, the CLSI M38 broth microdilution method was used to confirm their MIC values.

Table 2 shows the MICs determined by the CLSI method and the comparison of their values with those of YeastOne^TM^. Three isolates (CGMHD 1497, CGMHD 1524, and CGMHD 2417) showed high MIC values for AMB by YeastOne^TM^ and had lower values of 0.5–1 μg/mL by CLSI. The difference was up to three 2-fold dilutions. The MIC values of VRC determined by the CLSI methods were generally lower than those by YeastOne^TM^ (0.25–4 μg/mL vs. 0.5–8 μg/mL). However, the difference was within one 2-fold dilution. The MIC values of ITC by the CLSI methods were higher than those by YeastOne^TM^ (0.125–>16 μg/mL vs. 0.06–>16 μg/mL), and the difference was as high as three 2-fold dilutions. The MIC values of POS determined by CLSI methods were also higher than those from YeastOne^TM^ (0.125–0.5 μg/mL vs. 0.03–0.5 μg/mL) and the difference was two 2-fold dilutions. CGMHD 1652 and CGMMHD 2261 are voriconazole-resistant strains according to the clinical breakpoint (CBP) of 2 μg/mL defined by CLSI M61 2nd edition [23]. The resistance rate in this study was 1.77%. Both strains also had high MIC values for ITC (>16 μg/mL) and ISA (≥4 μg/mL). The isolate CGMHD 0744 was intermediately susceptible to VRC, with an MIC value of 1 μg/mL.

### 3.3. Detection of cyp51A Mutations in Azole-Resistant Isolates

The *cyp51A* gene of two azole-resistant strains and one intermediately susceptible strain was sequenced to detect mutations. Three different mutation patterns were detected in these three strains. Two resistant strains showed TR_34_/L98H (CGMHD 1652) and TR_34_/L98H/S297T/F495I (CGMHD 2261) mutations in *cyp51A*. The strain CGMHD 0744, which had intermediate susceptibility to VRC, had no tandem repeat in its *cyp51A* promoter region but had five amino acid substitutions (F46Y/M172V/N248T/D255E/E427K) due to point mutations in its coding regions.

### 3.4. Clinical Profiles of Patients from Whom the Azole-Resistant A. fumigatus Were Isolated

The clinical profiles of three patients, from whom azole-resistant *A. fumigatus* were isolated, are summarized in Table 3. CGMHD 1652 was isolated from the bronchoalveolar lavage of a patient diagnosed with bronchiectasis and bronchiolitis with symptoms of hemoptysis. The patient did not receive antifungal agents and had a favorable outcome. It is likely that the isolate was colonized and did not result in infection clinically. CGMHD 2261 was obtained from the bronchoalveolar lavage of a patient diagnosed with invasive fungal tracheobronchitis with a serum galactomannan value of 0.82. The patient died of respiratory failure and multi-organ failure, despite systemic VRC therapy. CGMHD 0744 was isolated from the bronchoalveolar lavage of a patient with necrotizing pneumonia caused by *Klebsiella pneumoniae* and pulmonary aspergillosis. The patient received systemic treatment with CAS but died of profound shock and multi-organ failure. The last two cases were considered as azole-resistant and intermediately susceptible strains related mortalities.

## 4. Discussion

Invasive aspergillosis (IA) is the most common invasive fungal infection in hematopoietic stem cell transplant recipients [24]. Other patient groups at risk of IA include those with prolonged neutropenia, cellular immunity deficiency, and those receiving immunosuppressive therapy, especially patients with graft-versus-host disease. In addition, increasing the use of biologics with tumor necrosis factor blockers, B cell-targeting monoclonal antibody (anti-CD20), and anti-rejection monoclonal antibodies (anti-CD52, CD25) have been associated with cases of IA [25].

The emergence of drug resistance in *Aspergillus* is an alarming issue in clinical practice because it may result in treatment failure or breakthrough infection, leading to patient mortality. Fungi can evolve different strategies to develop drug resistance. c*yp51A* encodes a key enzyme in the biosynthesis of ergosterol, and the Cyp51A protein is known as the target of azoles. The mutation of *cyp51A* caused altered affinity for azoles and mainly contributed to azole resistance [26]. The overproduction of the Cyp51A protein also led to the need for elevated effective drug concentrations and consequently resulted in azole resistance [27]. Resistance to polyenes has been reported with geographic specificity. Prevalence rates of AMB-resistant isolates have been reported to be 27% in Brazil and up to 94% in Hamilton, Canada [28,29]. Although still largely unknown, Ashu et al. proposed that the mechanism may be similar to the intrinsic resistance to AMB of *A. terreus*, associated with the upregulation of ergosterol biosynthesis genes and increased the expression of superoxide dismutase and catalase-encoding genes [29,30]. Decreased echinocandin susceptibility of *A. fumigatus* related to anidulafungin exposure was reported recently [31,32]. Mutations on *FKS1*, which encode the drug target β-1, 3-D-glucan synthase, are reported as the main mechanism. This results in the decreased sensitivity of glucan synthase to echinocandins and decreased echinocandin susceptibility and was associated with treatment failure in the case of chronic pulmonary aspergillosis with an aspergilloma [31,33].

Although isolates resistant to multiple azoles have been increasingly reported, CLSI did not establish CBPs for antifungal activity against *A. fumigatus* until the latest version of the 2nd edition of the CLSI M61 Performance Standards for Antifungal Susceptibility Testing of Filamentous Fungi was published in 2020 [23]. In this edition, the CBP of VRC is 2 μg/mL. Isolates were classified as susceptible, intermediate, and resistant if they had a VRC MIC value of <1 μg/mL, 1 μg/mL, and ≥2 μg/mL, respectively. This is the only antifungal drug for which CBP is defined in the CLSI. For other antifungals, the epidemiological cutoff values (ECVs) for *A. fumigatus* are provided in the 3rd edition of the CLSI M59 document [34]. The European Committee on Antimicrobial Susceptibility Testing (EUCAST) v. 10.0, also updated CBPs for ISA, ITC, VRC, and POS against *A. fumigatus* in 2020 [35]. Due to the different methodologies employed by CLSI and EUCAST, the interpretation of MIC values as susceptible or resistant should adhere to the protocol used. In this study, isolates CGMHD 1652 and CGMHD 2261 showed pan-azole resistance to VRC, ITC, and ISA, while CGMHD 0744 only showed elevation of VRC MIC but did not reach the resistance criteria.

Several mechanisms for *cyp51A*-mediated resistance have been reported including single point mutations (SNPs), multiple point mutations, and tandem repeats with or without point mutations. Some hotspot single-point mutations contribute to different phenotypes of resistance. These hotspots included G54, M220, G138, and G448. Most of these strains were resistant to multiple azoles [6,13]. Previous reports have proposed that these mutations may be associated with blocking the entry or modifying the binding site of the azoles, which further cause drug affinity reduction [14]. The combination of multiple SNPs, which cause amino acid substitutions, is another resistance mechanism. The most frequently reported mutations were the F46Y/M172V/D255E and F46Y/M172V/N248T/D255E/E427K substitutions. Strains possessing these two mutations have been reported to have different degrees of azole susceptibility. Even when regarded as “susceptible”, strains with these mutations generally showed higher MIC values for azoles compared to wild-type strains [13]. The susceptibility test of CGMHD 0744, which had five substitutions, only showed an elevation of the VRC MIC value, suggesting a marginal effect of this mutation. In a study of Cyp51A protein homology models, M172V, N248T, D255E, and E427K were nonsynonymous mutations that were located in non-conserved areas on the surface of the protein. Therefore, they were predicted to not interact with azole compounds or affect their structural integrity. In contrast, the substitution F46Y affected the transmembrane domain and substrate access channel of Cyp51A. Thus, the F46Y mutation was believed to be partially or totally responsible for the slightly higher azole MICs that these strains showed, due to the potential block of the substrate entrance channel [9]. In addition to point mutations, a TR in the promoter region, resulting in the overexpression of *cyp51A*, was related to azole resistance. Strains with TR or point mutations (L98H or Y121F) alone were found to have only a moderate increase in the MIC of the azoles. Strains with a combination of TR and amino acid substitutions can achieve significant azole resistance [27].

According to Wu et al. in 2020, the prevalence of azole-resistant isolates from 11 hospitals in Taiwan from 2011 to 2018 was 5.1% (19 of 375 isolates) [17]. The prevalence of azole-resistant isolates in our study was 1.77% (two out of 113 isolates). These two reports included the largest secondary and tertiary referral hospitals in Taiwan. Combining these two reports, the overall prevalence rate of the azole-resistant isolates was 4.3%. Among these azole-resistant isolates, six isolates had the TR_34_/L98H mutation, eight isolates had the TR_34_/L98H/S297T/F495I mutation, one isolate had the TR_46_/Y121F/T289A mutation, five isolates had the F46Y/G89G/M172V/N248T/D255E/L358L/E427K/C454C polymorphism, and one isolate had the F46Y/M172V/N248T/D255E/E427K polymorphism in the *cyp51A* gene. Another study by Chen et al. in 2019 reported that seven of 22 isolates of *A. fumigatus* from the environment were azole-resistant. Another two isolates of *A. fumigatus* isolated from patients in the study were azole-susceptible. Among the azole-resistant environmental isolates, three isolates had a TR_34_/L98H/S297T/F495I mutation, two isolates had TR_34_/L98H mutation, and two isolates hadTR_34_/L98H/S297T/F495I in the *cyp51A* gene [36]. Since most isolates were from the environment, we did not include the data from Chen et al. for the calculation of the clinical prevalence rate. The most prevalent two mutations reported by publications from Taiwan, TR_34_/L98H and TR_34_/L98H/S297T/F495I, which were found to be prevalent in the environment, are related to azole fungicide use in agriculture [37]. This was also proposed to be associated with the increasing use of azole fungicides (mainly difenoconazole, tebuconazole, and propiconazole) in Taiwan over the last two decades [8,17].

Azole-resistant *A. fumigatus* isolates have been reported globally, with prevalence varying from less than 1% to up to 28% based on different geographic regions [11,38]. The major prevalence of azole-resistant isolates was reported in European countries, with the highest prevalence in the United Kingdom [11,13]. In Asia, lower rates of azole resistance have been reported in Taiwan, Japan, China, and India [11,13]. Most of the reported resistance rates in these countries are lower than 10%. The overall resistance rate and related mutation mechanisms in Taiwan were similar to reports from other Asian countries [11,15,39,40]. However, the resistance rate varies between countries and also within a single country [13,41]. The variation in the prevalence of resistance may result from the geographic location, difference in laboratory practice, study design, and the diseases enrolled in each study [41]. A higher prevalence of azole-resistant isolates was found in some patients with specific risk factors such as previous prolonged azole exposure. Singh et al. reported that the resistance rate of isolates from patients with chronic pulmonary aspergillosis was up to 59% [42]. The high percentage of resistant isolates was probably linked to previous ITC exposure, which may be encountered in patients with aspergilloma, chronic aspergillosis, cystic fibrosis, and predisposing lung cavities [43]. Previous reports have also found that patients with hematologic or oncologic disease were more likely to harbor azole-resistant isolates, which cause invasive aspergillosis and were associated with high mortality [44,45].

According to the updated practice guidelines of the Infectious Diseases Society of America (IDSA) in 2016, the primary treatment for most invasive aspergillosis remains to be VRC, while AMB and ISA are alternative choices. Combination antifungal therapy with VRC and echinocandin may be considered in select patients [46]. Case 1 with the CGMHD 1652 strain received no antifungal therapy and had a favorable outcome. Case 2 with the CGMHD 2261 strain received VRC treatment as per the guidelines. The MIC of VRC for this isolate was 2 μg/mL as determined using the CLSI method, and this isolate was resistant to VRC. No susceptibility data were available at that time, thus, no alternative or combined antifungal agents were used. The patient had poor treatment outcomes and died of respiratory failure. Case 3 with the CGMHD 0744 strain was infected with an intermediate azole-susceptible isolate and treated with CAS because of an underlying condition of liver failure. Although the MEC of CAS was low, the patient died of profound shock and multi-organ failure. None of the patients received a combination of antifungal agents. However, in a randomized, double-blind multicenter study, mortality rates were higher in patients diagnosed with invasive aspergillosis receiving monotherapy with VRC than in those receiving combined therapy with AMB [47]. Alternative therapeutic agents and/or combined therapy should be considered in countries with azole-resistant strains or in patients with poor treatment response. Antifungal susceptibility tests should also be considered and might be helpful for the choice of alternative agents.

The MIC values determined by YeastOne^TM^ and CLSI slightly differed in this study. Generally, the MIC values of AMB and VRC obtained from YeastOne^TM^ were higher than those from CLSI. The MIC values of ITC and POS obtained by YeastOne^TM^ were lower than those obtained by CLSI. Similar observations were also noted in previous publications [48,49,50]. Likewise, higher MICs of AMB of some isolated by YeastOne^TM^ in this study were in fact wild type strains by CLSI. Thus, although YeastOne^TM^ had a good performance of an overall agreement of more than 90% with CLSI when testing the *Aspergillus* species [48], confirmation with CLSI or EUCAST to identify resistant isolates was necessary when the MIC levels of ITC, VRC, POS, and AMB were close to CBP or ECV.

There are several cryptic species in the *A. fumigatus* species complex such as *A. lentulus*, *A. novofumigatus*, *A. fischeri*, *A. viridinutans*, and *A. udagawae*, which may be intrinsically resistant to one or more antifungal agents [51,52]. According to a multicenter study from 19 countries, the overall prevalence of azole resistance was 3.2% among 2941 *A. fumigatus* species complex isolates. However, up to 21.7% of the azole-resistant isolates were not identified as *A. fumigatus*, but as cryptic species such as *A. lentulus*, *A. thermomutatus*, and *A. udagawae* [53]. This finding emphasizes the importance of the correct molecular identification of species when treating *Aspergillus* infections, especially invasive aspergillosis. Although some isolates demonstrated atypical morphology such as pale colony color or delayed sporulation, we did not find any antifungal-resistant cryptic species in this study using sequence-based identification.

## 5. Conclusions

Azole-resistant isolates of *A. fumigatus* are present in CGMH and all over Taiwan. TR_34_/L98H and TR_34_/L98H/S297T/F495I are the most prevalent *cyp51A* mutations based on current publications and this study. Accurate species identification with aggressive antifungal susceptibility plays an important role in the choice of antifungal agent. YeastOne^TM^ is a rapid and useful clinical tool for the antifungal susceptibility test. Although high agreement between YeastOne^TM^ and the CLSI method was reported, a discrepancy between the results is still possible. When utilizing YeastOne^TM^ in a clinical setting, the results should be interpreted carefully, and further confirmation by CLSI should be always considered when the MIC levels of VRC, POS, and AMB were close to CBP or ECV. Modification of clinical treatment strategies based on susceptibility testing is necessary when encountering azole-resistant *A. fumigatus*, which may be helpful to prevent morbidity and mortality.

## Figures and Tables

**Table 1:** The MEC and MIC data of nine antifungal agents using the YeastOne^TM^ method (μg/mL).

	AFG	MFG	CAS	5FC	POS	VRC	ITC	FLC	AMB
MIC range	≤0.015	≤0.008	≤0.008–0.06	8–>64	≤0.008–0.5	0.25–8	≤0.015–>16	32–>256	1–4
MIC_50_	≤0.015	≤0.008	≤0.008	>64	0.03	0.5	0.06	>256	2
MIC_90_	≤0.015	≤0.008	0.015	>64	0.03	0.5	0.12	>256	2

Abbreviations: AFG—anidulafungin, MFG—micafungin, CAS—caspofungin, 5FC—5-flucytosine, POS—posaconazole, VRC—voriconazole, ITC—itraconazole, FLC—fluconazole, AMB—amphotericin B.

**Table 2:** The MIC values (μg/mL) of different antifungals against seven *Aspergillus fumigatus* isolates as determined by the YeastOne^TM^ and CLSI methods.

Strain No.	AFST Method	ISA	POS	VRC	ITC	FLC	AMB
CGMHD 1497	YeastOne	ND	0.03	0.5	0.06	>256	4
	CLSI	0.25	0.125	0.25	0.25	>64	0.5
CGMHD 1524	YeastOne	ND	0.03	0.5	0.06	>256	4
	CLSI	0.5	0.125	0.25	0.5	>64	0.5
CGMHD 2417	YeastOne	ND	0.03	0.5	0.06	>256	4
	CLSI	0.25	0.125	0.25	0.125	>64	1
CGMHD 0641	YeastOne	ND	0.03	1	0.12	>256	1
	CLSI	0.5	0.125	0.5	0.25	>64	0.25
CGMHD 0744	YeastOne	ND	0.06	1	0.12	>256	2
	CLSI	1	0.25	1	0.5	>64	0.5
CGMHD 1652	YeastOne	ND	0.5	8	>16	>256	2
	CLSI	4	0.5	4	>16	>64	0.25
CGMHD 2261	YeastOne	ND	0.5	1	>16	>256	2
	CLSI	>4	0.5	2	>16	>64	1

AFST—antifungal susceptibility testing; ND—not performed because isavuconazole is not included in the YeastOne^TM^ panel; CLSI—Clinical and Laboratory Standards Institute, M38-3rd Ed. Reference method for broth dilution antifungal susceptibility testing of filamentous fungi.

**Table 3:** The clinical profile of patients infected by resistant *Aspergillus fumigatus* and mycological characteristics of the strains.

Case No.	Age/ Gender	Specimen	Clinical Profile	Strain	*cyp51A* Mutation	MIC (μg/mL)			
						POS	VRC	ITC	ISA
Case 1	50/ female	BAL	**Clinical diagnosis:** Hemoptysis due to bronchiectasis and bronchiolitis **Treatment course:** Symptomatic treatment **Outcome:** The patient had a favorable outcome and visited as a regular outpatient for more than 3 years.	CGMHD 1652	TR_34_/L98H	0.5	4	>16	4
Case 2	82/male	BAL	**Clinical diagnosis:** Invasive fungal tracheobronchitis. GM index in serum was 0.82. **Treatment course:** Voriconazole 300mg q12h loading day 1 then voriconazole 200mg q12h **Outcome:** After 19 days of voriconazole treatment, the patient died of respiratory failure with multi-organ failure.	CGMHD 2261	TR_34_/L98H/S297T/F495I	0.5	2	>16	>4
Case 3	80/ female	BAL	**Clinical diagnosis:** Necrotizing pneumonia caused by carbapenem-resistant *Klebsiella pneumoniae*, and invasive aspergillosis. GM index in serum was 5.55. **Treatment course:** Caspofungin 35 mg qd due to liver failure **Outcome:** The patient died of profound shock, respiratory failure, and multi-organ failure after 10 days of treatment.	CGMHD 0744	F46Y/M172V/N248T/D255E/E427K	0.25	1	0.5	1

## Data Availability

Data presented in this study are available upon request from the corresponding author.
